# Invading Basement Membrane Matrix Is Sufficient for MDA-MB-231 Breast Cancer Cells to Develop a Stable *In Vivo* Metastatic Phenotype

**DOI:** 10.1371/journal.pone.0023334

**Published:** 2011-08-15

**Authors:** Mohamed Abdelkarim, Nadejda Vintonenko, Anna Starzec, Aniela Robles, Julie Aubert, Marie-Laure Martin, Samia Mourah, Marie-Pierre Podgorniak, Sylvie Rodrigues-Ferreira, Clara Nahmias, Pierre-Olivier Couraud, Christelle Doliger, Odile Sainte-Catherine, Nicole Peyri, Lei Chen, Jérémie Mariau, Monique Etienne, Gerard-Yves Perret, Michel Crepin, Jean-Luc Poyet, Abdel-Majid Khatib, Mélanie Di Benedetto

**Affiliations:** 1 Université Paris 13, CNRS FRE CSPBAT, Laboratoire de Chimie Structurale Biomoléculaire, UFR SMBH, Bobigny, France; 2 INSERM 553 Endothélium et Angiogénèse Laboratoire d'Hémostase, Paris, France; 3 Université Paris 13, EA4222, Li2P, Bobigny, France; 4 Université Paris 7, UMRS 940. Equipe Avenir, IGM, Paris, France; 5 AgroParisTech, UMR 518, Mathématiques et Informatique Appliquées, Paris, France; 6 URGV UMR INRA 1165-CNRS 8114-UEVE, Evry, France; 7 Inserm, U1016, Institut Cochin, Paris, France; 8 CNRS UMR 8104, Paris, France, Université Paris Descartes, Paris, France; 9 Service Commun d'Imagerie, Institut d'Hématologie, Hôpital Saint Louis, Paris, France; 10 Université Paris 13, Laboratoire d'Histologie, Bobigny, France; 11 INSERM U1029 Université Bordeaux 1, Bordeaux, France; Cornell University, United States of America

## Abstract

**Introduction:**

The poor efficacy of various anti-cancer treatments against metastatic cells has focused attention on the role of tumor microenvironment in cancer progression. To understand the contribution of the extracellular matrix (ECM) environment to this phenomenon, we isolated ECM surrogate invading cell populations from MDA-MB-231 breast cancer cells and studied their genotype and malignant phenotype.

**Methods:**

We isolated invasive subpopulations (INV) from non invasive populations (REF) using a 2D-Matrigel assay, a surrogate of basal membrane passage. INV and REF populations were investigated by microarray assay and for their capacities to adhere, invade and transmigrate *in vitro*, and to form metastases in nude mice.

**Results:**

REF and INV subpopulations were stable in culture and present different transcriptome profiles. INV cells were characterized by reduced expression of cell adhesion and cell-cell junction genes (44% of down regulated genes) and by a gain in expression of anti-apoptotic and pro-angiogenic gene sets. In line with this observation, *in vitro* INV cells showed reduced adhesion and increased motility through endothelial monolayers and fibronectin. When injected into the circulation, INV cells induced metastases formation, and reduced injected mice survival by up to 80% as compared to REF cells. In nude mice, INV xenografts grew rapidly inducing vessel formation and displaying resistance to apoptosis.

**Conclusion:**

Our findings reveal that the *in vitro* ECM microenvironment *per se* was sufficient to select for tumor cells with a stable metastatic phenotype *in vivo* characterized by loss of adhesion molecules expression and induction of pro-angiogenic and survival factors.

## Introduction

Metastasis in breast cancer patients accounts for over 90% of the deaths. Preclinical studies reveal that many drugs used in the management of primary tumors are not or less effective against metastasis [1]. Although the mechanism by which metastases develop is still not fully understood, it is generally believed that tumor cells acquire features that affect their metastatic potential during the progression of the tumor; these features include increased survival, invasive and migratory abilities. Metastasis is a complex cascade of sequential steps, none of which being fully understood. Many studies implicated the stroma in the development of metastases. Stroma and cancer cell interactions were found to contribute to cell detachment from primary tumors, intravasation into the blood stream, and extravasation at distant sites where tumor cells can seed and form tumor metastases [2]. Previously, fibroblasts, endothelial cells and macrophages and other stroma cells were reported to be implicated in the occurrence of metastases [2]–[4]. Whereas less is known about the influence of the extracellular matrix (ECM) in the development of metastases [5], [2], ECM appears to be involved, particularly through sequestration of soluble factors secreted by stroma cells and acting on tumor cells in a paracrine manner [6]–[9]. The ECM can modify the adhesion of cancer cells leading to their invasive behavior. However, it is not clear if the ECM, and particularly the basement membrane, can influence the selection of metastatic cells with a well-defined genotype profile. The basement membrane underlies the endothelium in the vessel wall and is the major barrier to tumor cell extravasation and invasion [10]–[11]. Matrigel is a basement membrane extract derived from a murine tumor [12]. The components of this tumor basement membrane are similar, both chemically and immunogically, to authentic basement membrane components [13]. Consequently, Matrigel may be used as an experimental model of barrier to identify both inhibitors and activators of invasion [14].

Most of previously reported studies on gene profiling in malignant cells used tumor cells isolated from metastatic foci that were already well established *in vivo*. Therefore, the contribution of ECM to the selection of metastatic cells has not been rigorously assessed. Here, we report the *in vitro* selection of two cell populations derived from MDA-MB-231 breast cancer cells [15], on the basis of their high (INV) invasive ability (to migrate through Matrigel). We examined the transcriptomes of these two populations using micro arrays and correlated their gene profiles to their malignant phenotypes.

## Materials and Methods

### Cell culture

Human breast adenocarcinoma MDA-MB-231 cells were obtained from the American Type Culture Collection (Manassas, VA, USA). The INV and REF cells isolated as described underneath were maintained in 10% FBS-DMEM, 1% sodium pyruvate with 1% penicillin and 1% streptomycin at 37°C in a humidified atmosphere containing 5% carbon dioxide. Human microcapillary brain endothelial hCMEC/D3 cells [16], [17] were grown in collagen type I-coated dishes in EBM-2 media supplemented with 2.5% FBS, hydrocortisone and growth factors (VEGF, IGF, EGF and bFGF EGM-2 Bullet kit, Cambrex-Biowhittaker).

### Isolation of MDA-MB-231 derived INV and REF variants

MDA-MB-231 cells (2.5×10^5^) were seeded in 8 µm-pore size Boyden chambers (Becton Dickinson) coated with Matrigel (Falcon, MA, USA) and incubated for 16 h at 37°C in a 5% CO2 atmosphere. Invading cells, referred to as INV cells, were collected from the bottom of the membrane in 0.5 mM EDTA and cultured in a 24-well plate. The selection was repeated eight times sequentially in order to obtain highly invasive cells. To select non-invasive cells (REF), the same procedure was used and the cells that remained on top of the membrane after the first round of selection were collected.

### Cell growth, migration and invasion assay

For cell growth assays, INV and REF cells were seeded at 2×10^5^ cells per well in 6-well plates and cultured for 72 h. Cell migration experiments were performed using the Boyden chambers as described above: the cells (2.5×10^5^) were put into the upper chamber and 10% FCS-DMEM was used as a chemoattractant. For invasion assays, chambers were coated with Matrigel as described above. Then, cells on the lower face of the filter were fixed, stained with crystal violet and counted under the optical microscope.

### Adhesion and transmigration assays

Tumor cells were harvested in 0.5 mM EDTA and seeded at 5×10^5^ cells per well in 96-well plates pre-coated with 1 µg/ml of fibronectin (Sigma, France) and incubated at 37°C for 1 h. The plates were washed with PBS, and adherent cells were stained with crystal violet/methanol (0.5%/20%) for 1 h at room temperature. The plates were again washed, and were incubated in ethanol: 0.1 M citrate (1∶1) pH 4.2 for 30 min at room temperature and absorbance was measured at 540 nm using an ELISA reader (Labsystem, GMI Inc, Minnesota, USA). To study tumor cell adhesion to endothelial cell monolayers, hCMEC/D3 cells were seeded at 10^4^ cells per well on collagen type I-coated 96-wells plates and left to grow until confluence. The cells were then starved for 48 h and incubated for an additional 24 h with the proinflammatory cytokines IFNγ (100 U/ml-Preprotech Inc, NJ, USA.) and TNFá (200 U/ml-Preprotech Inc.). Next, the INV and REF cells at 80% confluence were harvested using PBS-EDTA and labeled for 30 min at 37°C with CMFDA, a green fluorescent cell tracker (Invitrogen, Cergy Pontoise, France). Then, 10^5^ INV or REV cells were added to the endothelial cell monolayer and incubated for 30 min at 37°C. The unattached cells were removed and adherent cells were lysed with water. Fluorescence was measured at 485/530 nm using a microplate reader (Labsystem, France). For transmigration assays, Boyden chambers were coated with hCMEC/D3 endothelial cells and CMFDA-labeled tumor cells were counted by fluorescent microscopy.

### Gelatine zymography

INV and REF cells were cultured in serum-free media for 24 h in 6-well plates. Conditioned media were then lyophilized and normalized to the number of cells. They were mixed with non-reducing Laemmli buffer and run on a 10% SDS-polyacrylamide gel containing 1 mg/ml gelatin. The gel was washed three times at room temperature in a solution containing 2.5% (v/v) Triton X-100 and incubated at 37°C for 24 h in 50 mM Tris/HCL, pH 7.5, 0.2 M NaCl, 5 mM CaCl_2_ and 0.05% Brij 35. The gel was then stained for 60 min with 0.25% (w/v) R-250 Coomassie blue in 45% (v/v) methanol/10% (v/v) acetic acid and destained in 25% methanol (v/v)/10% acetic acid (v/v).

### Cell death detection

Cells (2×10^5^) were cultured for 96 h in a serum-free medium or with 2.5 µM Doxorubicin for 24 h in a serum-containing medium. Then, cells were harvested and apoptotic cells were labeled using the Annexin V-FITC Apoptosis Detection kit (Beckman coulter, Fullerton CA, USA). The samples were then analyzed by flow cytometry (Becton Dickinson, Heidelberg, Germany). For detection of cell death *in vivo*, tumor sections (5 mm) were deparaffinized and rehydrated, then analyzed for DNA fragmentation using a TumorTACS kit (R&D Systems, Abington, UK). Various fields (10 per section) were selected for analysis and the intratumor apo-necrotic cells were counted as previously described [18]. For AKT phosphorylation assay, cells were treated for 1 h or 72 h with serum free medium and stimulation with IGF-1 at 10 ng/ml for 30 min was used as positive control.

### Western blots

INV and REF cell lysates were clarified by centrifugation, and protein concentrations were measured by bicinchoninic acid protein assay (Bio-Rad, Marne-La-Coquette, France). Equal amounts of total proteins were resolved by 6% SDS-PAGE and transferred to nitrocellulose membrane. The membrane was blocked in 5% nonfat dried milk in Tris-buffered saline containing 1% Tween 20 and incubated with primary antibodies against NRP-1 (Santa-Cruz Biotechnologies inc., CA) or ß-actin (Sigma, Saint quentin Fallavier, France), AKT or pAKT (Cell signaling technology, Boston, USA) for 1 h, and then with an appropriate conjugated secondary antibody for 30 min at room temperature. Immunoreactive bands were detected by chemiluminescence according to the manufacturer's instructions.

### VEGF quantification

INV or REF cells seeded in 24-well culture plates (2×10^5^/well) in DMEM–10% FCS were allowed to adhere for 24 h. Conditioned media containing growth factors secreted by the cells were collected after 48 h of serum starvation. The VEGF content in these media was measured by quantitative enzyme-linked immunoabsorbent sandwich assay (ELISA) using the Quantikine kit (R&D Systems, Abington, UK) according to the manufacturer's instructions. VEGF contents were estimated from a standard curve established from serial dilutions of VEGF protein standards (R&D Systems). The values reported for VEGF secretion are normalized to cell number.

### Real-time RT-PCR analysis

Total RNA (1 µg) was reverse-transcribed using MMLV RT (Invitogen, Carlsbad, CA, USA) and random primers from Roche Applied Science (Roche, Indianapolis, IN, USA). Primers for qRT-PCR were purchased from AnyGenes (Creteil, France). Real-time PCR was carried out with the LightCycler® 2.0 Real-Time PCR System (Roche, Indianapolis, IN, USA) using the LightCycler® FastStart DNA Master^PLUS^ SYBR Green I Kit. The reactions were cycled 45 times [95°C, 10 min, (94°C, 15 sec; 60°C, 1 min; and 72°C, 1 min)]. Reported results are normalized to the expression of the *PPIA*, *GAPDH*, *TBP*, *beta 2 Microglobin*, *Actin*, *RPL0* and *RPL19* genes that exhibit little variation in this data set. Similar results were obtained for all reference genes, the representative data is normalized to *PPIA*. Fold induction relative to internal controls was calculated by the delta Ct evaluation method [19].

### Gene Expression Profiling

Total RNA was isolated from eight cell cultures (four biological replicates corresponding to four different passages of both INV and REF cells) using the RNeasy mini kit (Qiagen, Valencia, CA, Germany) by direct cell lysis in 10 cm culture dishes containing 600 µl RLT Buffer following the manufacturer's instructions. Purified RNA was quantified with a Nanodrop ND-1000 spectrophotometer (Thermo Fisher Scientific, Hudson, NH, USA) and its quality was evaluated by running the Eukaryotic Total RNA Nano Assay in the Bioanalyser 2100 (Agilent Technologies, Chandler AZ, USA) using the Agilent RNA 6000 Nano Kit. Probes (300 ng of the total RNAs) were prepared for hybridization to Affymetrix GeneChip® Human Gene 1.0 ST arrays according to the manufacturer's instructions. After scanning, eight CEL files generated by GCOS (Affymetrix GeneChip® Operating Software) were uploaded into Expression Console Software (Affymetrix, Santa Clara, CA, USA) for normalization and probe set summarization using the RMA (Robust Multichip Analysis) algorithm [20]. The resulting data in terms of probe set intensities were exported into a .txt file for further statistical analysis. The microarray data has been deposited in the Gene Expression Omnibus (GEO) database at http://www.ncbi.nlm.nih.gov/geo/ with the accession number GSE16838.

### Microarray data analysis

Analyses were carried out in R, a free software environment available at http://www.r-project.org/. To identify differentially expressed genes, differences between the two conditions were calculated on each day of measurement and then analyzed using a double-sided paired t-test implemented in the anapuce R package (http://www.agroparistech.fr/mia/outil.html). Pairing was carried out to remove any source of variation due to the day of measurement from the comparison of the two conditions. The variance was split between subgroups of genes with homogeneous variance [21]. Statistical raw p-values were adjusted for multiple comparisons using the Benjamini-Hochberg procedure [22] which controls the False Discovery Rate (FDR) after the estimation of the proportion of genes non-differentially expressed using the smoother method [23]. The level of statistical significance was set at 0.05 for all comparisons. Gene annotations and functions were assessed using NetAffx™ Analysis Center (Affymetrix, Santa Clara, CA, USA), Ingenuity pathway Analysis (IPA) software (Ingenuity SystemsInC.) and data mining.

### Xenografts in nude mice

All in vivo experiments were carried out with the approval of Département d'Expérimentation Animale, Institut Universitaire d'Hématologie, Hôpital Saint-Louis ethical committee (permit number 75516) and according to the UKCCCR guidelines. The animals were kept in a temperature-controlled room on a 12∶ 12 light-dark schedule with food and water *ad libidum*. Aliquots of 2×10^6^ of INV (n = 7) or REF cells (n = 7) were inoculated subcutaneously (s.c) near the mammary gland of 4-week-old athymic nude mice (nu/nu, Janvier, France, n = 14). Tumor volume was measured once a week along two major axes using calipers. Tumor volume (mm^3^) was calculated as follows: V = 4/3πR_1_^2^R_2_ where R_1_<R_2_

### Immunohistochemical analysis

Tumor specimens were fixed with 4% paraformaldehyde and embedded in paraffin using standard procedures. For immunohistochemical studies, 5 µm-thick sections were cut, deparaffinized and rehydrated. Routinely, sections were stained in haematoxylin and eosin. Endogenous peroxidase was inactivated with 3% H_2_O_2_ and washed in TBS (0.05 M Tris, 1.5 M NaCl, pH 7.6) followed by preincubation in 10% normal goat serum for 1 h at room temperature. Endothelial cells were specifically labeled with GSL-1 isolectin B4 (Vector Laboratories, Burlingame, CA, U.S.A.) as described previously [24]. For each tumor, 10 randomly selected sections were analyzed.

### Intracardiac experimental metastasis assay

We performed mice intracardiac injection of bioluminescent INV and REF cells as also described previously in order to observe large dissemination behavior of each subpopulation [25]. For this study, luciferase-expressing INV and REF cell pools were obtained by stable transfection with a plasmid carrying a G418 resistance gene and the luciferase gene (pEGFPLuc, BD Biosciences, Rungis, France) using Fugen-6 (Roche Diagnostics, Meylan, France). Transfected cells were cultured in 10% FCS-DMEM supplemented with G418 at 1 mg/ml. For experimental metastasis, female nude mice (8–10 weeks) were anesthetized by intraperitoneal injection with 120 mg/kg ketamine and 6 mg/kg xylazine on the day of cell injection or by exposure to 1–3% isoflurane on subsequent imaging days. pEGFPLuc-transfected INV or REF cells (10^5^) in 100 µl of sterile DPBS were injected into the left ventricle of the heart by nonsurgical means. Anesthetized mice were given intraperitoneal injections of D-luciferin (Caliper Life Science) and placed in the IVIS™ Imaging System (Xenogen, Caliper Life Science, MA, USA) and imaged from both dorsal and ventral views. Successful intracardiac injection was revealed on day 0 by systemic bioluminescence distributed throughout the whole animal. Only mice with satisfactory injection were used for subsequent experiments. Metastasis analysis was monitored by *in vivo* imaging once a week for up to eight weeks.

### Bioluminescent Imaging

Bioluminescence images were acquired with the IVIS imaging system (Xenogen) 5 minutes after intraperitoneal injection of D-luciferin into the anesthetized animals. Acquisition time at the beginning of experiment was 5 min and was reduced in keeping with signal strength to avoid saturation. Analysis was performed using LivinImage software (Xenogen) by measurement of photon flux (photon/s/cm^2^) within a region of interest (ROI) drawn around the bioluminescence signal to detect the metastatic sites.

### Statistical analysis

Student's t-test was used for statistical analysis. P<0.05 was considered significant.

## Results

### Selection of INV subpopulation from MDA-MB-231

**Figure 1:**
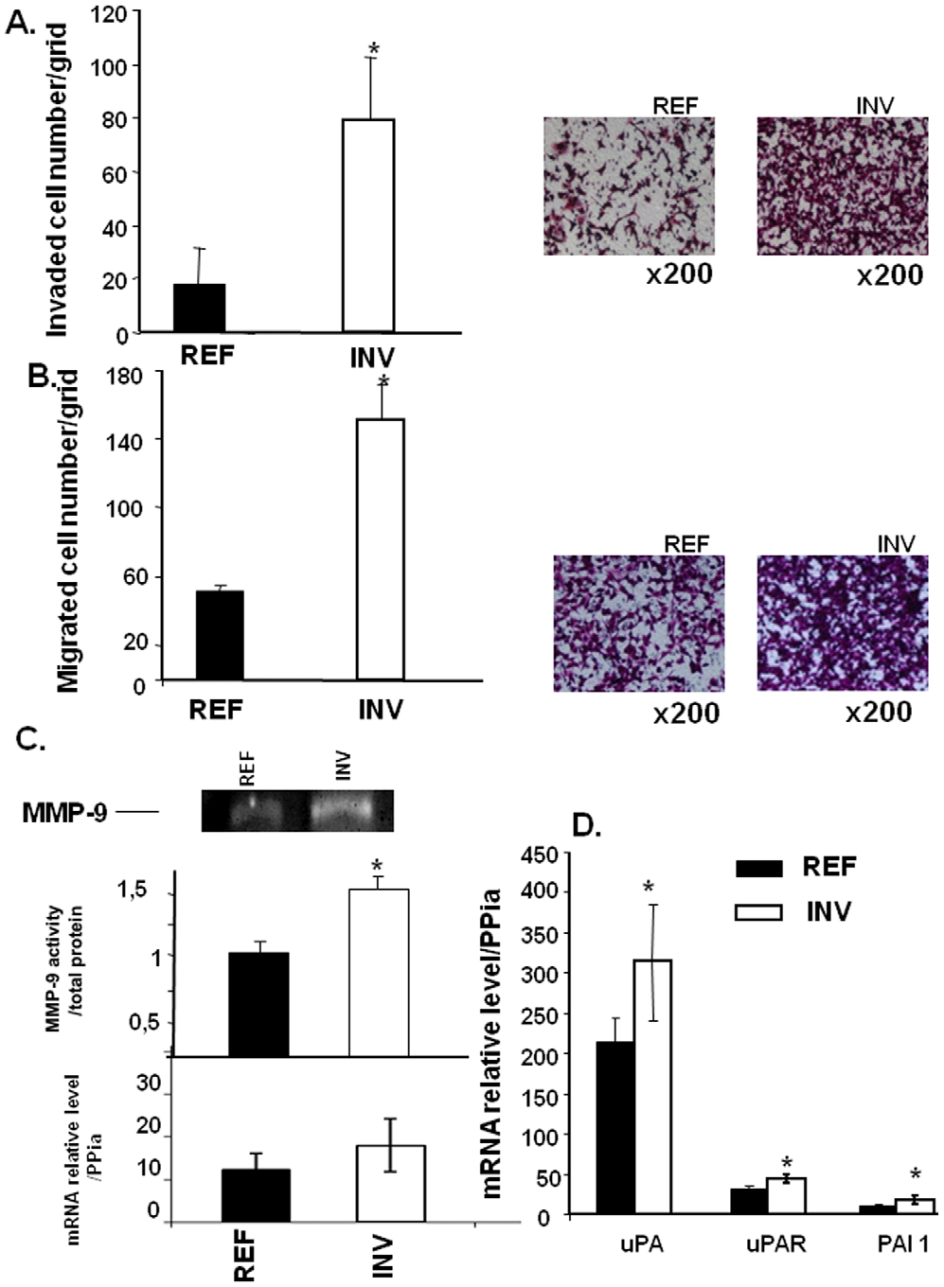
INV and REF breast cancer cell invasion and migration. Cells (2.5×10^5^) were added to the upper side of each 8 µm insert of Boyden chambers coated (A) or not (B) with Matrigel for invasion or migration assays respectively; cells were then counted as described in Materials and Methods. MMP-9 activity secreted by REF (lane 1) and INV (lane 2) cells (C) was assessed by subjecting aliquots of lyophilized conditioned media normalized to the number of cells to 10% SDS-polyacrylamide gels containing 1 mg/mL gelatin. For QRT-PCR analysis (D), total RNA (1 µg) was reverse-transcribed using MMLV RT and subjected to qRT-PCR as described in Material and Methods. Representative data was normalised to *PPIA* is given. Each column represents a mean (± SD) of three independent experiments. *P<0.05.

Using Matrigel coated Boyden chambers, we established two different sub-populations of MDA-MB-231 cells. These included the invasive MDA-MB-231 subpopulation (INV) that was isolated from a less invasive subpopulation (REF) following eight rounds of invasion assays in the Boyden Chamber. Their invasive phenotype was confirmed by migration and invasion assays (Fig. 1). REF cells were expanded and frozen after the first round. As expected, INV cells were up to 4-fold more invasive than REF cells (Fig. 1A and right panel), and up to 3-fold more motile (Fig. 1B and right panel). These properties were stable and not lost after maintenance in culture or freezing. To assess the invasive capacity of INV and REF cells in more detail, the corresponding conditioned media were collected and analyzed for gelatinase enzymatic activity (Fig. 1C). MMP-9 activity was higher in media conditioned by INV cells as compared to REF cells. In contrast, MMP-2 activity remained unchanged (data not shown). Similarly, the mRNA expression of uPA, uPAR and PAI-1 were found significantly up-regulated in INV cells as evaluated by real time PCR (Fig. 1D).

### Transcriptomic analysis of INV and REF cells

We used the Affymetrix Human Gene 1.0ST arrays that contain 28000 annotated genes (Table 1 and 2), to investigate the expression profile of REF and INV cells. Comparative analysis of REF and INV cells revealed 134 differentially expressed genes (Table S1): 38 were down-regulated and 96 were up-regulated in INV cells with respect to REF cells. Among the known down-regulated genes in INV cells (Table 1), 44% are involved in cell adhesion and cell-cell junctions, and 22% in induction of apoptosis and G-protein coupled receptor signaling (Fig. S1). Of the up-regulated genes in INV (Table 2) with known function, 25% are involved in survival via negative regulation of apoptosis, 17.5% in cytoskeleton reorganization, cell proliferation and chemotaxis, 12.5% in cell adhesion and cell-cell junctions and 10% in G-protein coupled receptor signaling (Fig. S1).

**Table 1:** List of genes that are down-regulated in INV cells *versus* REF cells.

Gene Symbol	Gene Title	Description	log fold	Adjusted p-value
CCND2*	cyclin D2	cell cycle	−2,25	0,039
FERMT3*	fermitin family homolog 3 (Drosophila)	cell adhesion	−1,70	0,039
SYTL3	synaptotagmin-like 3	intracellular protein transport /// Rab GTPase binding	−1,61	0,021
GABRA3	gamma-aminobutyric acid (GABA) A receptor, alpha 3	ion transport /// cell junction	−1,38	0,022
ABI3	ABI gene family, member 3	regulation of cell migration	−1,28	0,039
HMGCS1	3-hydroxy-3-methylglutaryl-Coenzyme A synthase 1 (soluble)	isoprenoid biosynthetic process	−1,16	0,046
OCLN	occludin	cell junction	−1,12	0,040
GPR176	G protein-coupled receptor 176	G-protein coupled receptor protein signaling pathway	−1,07	0,040
AMIGO2	adhesion molecule with Ig-like domain 2	cell adhesion /// negative regulation of programmed cell death	−1,03	0,046
GNGT2	guanine nucleotide binding protein (G protein), gamma transducing activity polypeptide 2	signal transduction /// G-protein coupled receptor protein signaling pathway	−0,99	0,039
ASAM	adipocyte-specific adhesion molecule	cell junction	−0,97	0,042
HMGCR	3-hydroxy-3-methylglutaryl-Coenzyme A reductase	isoprenoid biosynthetic process	−0,93	0,050
SCARF1	scavenger receptor class F, member 1	cell adhesion	−0,91	0,039
STAT5A	signal transducer and activator of transcription 5A	transcription factor activity /// positive regulation of cell proliferation /// regulation of cell adhesion /// regulation of epithelial cell differentiation /// negative regulation of apoptosis /// positive regulation of survival gene product expression /// positive regulation of mitotic cell cycle /// positive regulation of inflammatory response	−0,81	0,047
CASP4*	caspase 4	induction of apoptosis	−0,79	0,046
PCDHB2	protocadherin beta 2	cell adhesion	−0,74	0,040
CARD6	caspase recruitment domain family, member 6	regulation of apoptosis	−0,73	0,043
TP53BP1	tumor protein p53 binding protein 1	DNA repair /// transcription activator activity	−0,72	0,046

Genes' functions are based on GO Description provided by Affymetrix, NetAffx data base (https://www.affymetrix.com/analysis/netaffx/index.affx).

*Validated by QRT-PC.

**Table 2:** List of genes that are up-regulated in INV cells *versus* REF cells.

Gene Symbol	Gene Title	Description	log fold	Adjusted p-value
MID2	midline 2	cytoskeleton /// microtubule associated complex	0,58	0,049
PLP2	proteolipid protein 2 (colonic epithelium-enriched)	chemotaxis	0,61	0,048
TGFB2*	transforming growth factor, beta 2	angiogenesis /// epithelial to mesenchymal transition /// cell growth /// extracellular matrix organization and biogenesis /// regulation of apoptosis /// positive regulation of cell cycle /// somatic stem cell division /// negative regulation of epithelial cell proliferation /// regulation of immune response	0,62	0,048
CETN2	centrin, EF-hand protein, 2	cell cycle /// mitosis /// cell division	0,64	0,046
TMSB4X /// TMSL1,2,3,6	thymosin beta 4, X-linked /// thymosin-like 1,2,3 and 6 (pseudogenes)	cytoskeleton organization and biogenesis	0,64	0,046
GPR17	G protein-coupled receptor 17	chemokine receptor activity /// G-protein coupled receptor protein signaling pathway	0,65	0,042
L1CAM*	L1 cell adhesion molecule	cell adhesion /// cell differentiation	0,66	0,046
GDI1	GDP dissociation inhibitor 1	Rab GDP-dissociation inhibitor activity /// small GTPase mediated signal transduction	0,69	0,045
MAGED1	melanoma antigen family D, 1	transcription coactivator activity /// regulation of apoptosis /// negative regulation of epithelial cell proliferation	0,70	0,048
RBBP7*	retinoblastoma binding protein 7	cell proliferation	0,70	0,042
CD99L2	CD99 molecule-like 2	cell adhesion /// cell junction	0,71	0,040
ATP6AP2	ATPase, H+ transporting, lysosomal accessory protein 2	angiotensin maturation /// receptor activity /// positive regulation of transforming growth factor-beta1 production /// regulation of MAPKKK cascade	0,73	0,040
FANCB	Fanconi anemia, complementation group B	DNA repair	0,73	0,040
IRAK1*	interleukin-1 receptor-associated kinase 1	signal transduction /// transmembrane receptor protein serine/threonine kinase signaling pathway /// activation of NF-kappaB-inducing kinase activity /// transcription activator activity /// interleukin-1 receptor complex	0,73	0,042
SUV39H1	suppressor of variegation 3–9 homolog 1 (Drosophila)	cell cycle /// cell differentiation	0,73	0,040
BCAP31	B-cell receptor-associated protein 31	apoptosis /// immune response	0,74	0,040
FGD1	FYVE, RhoGEF and PH domain containing 1	cytoskeleton organization and biogenesis /// signal transduction /// regulation of cell shape /// small GTPase binding /// regulation of Rho protein signal transduction	0,74	0,040
NKAP	NFKB activating protein		0,75	0,040
RAB9A	RAB9A, member RAS oncogene family	small GTPase mediated signal transduction /// protein transport	0,77	0,040
CD99	CD99 molecule	cell adhesion	0,77	0,042
ARAF	v-raf murine sarcoma 3611 viral oncogene homolog	protein serine/threonine kinase activity	0,78	0,040
AIFM1	apoptosis-inducing factor, mitochondrion-associated, 1	DNA fragmentation during apoptosis /// apoptosis /// DNA damage response, signal transduction resulting in induction of apoptosis	0,79	0,040
HCLS1	hematopoietic cell-specific Lyn substrate 1	transcription factor activity /// positive regulation of cell proliferation /// response to hormone stimulus /// positive regulation of tyrosine phosphorylation of STAT protein	0,79	0,049
HDAC6	histone deacetylase 6	cell cycle /// specific transcriptional repressor activity	0,79	0,039
APEX2	APEX nuclease (apurinic/ apyrimidinic endonuclease) 2	DNA repair /// lyase activity	0,79	0,040
UXT	ubiquitously-expressed transcript	microtubule cytoskeleton organization and biogenesis	0,80	0,045
SLC25A6	solute carrier family 25, member 6	mitochondrial transport /// apoptosis	0,83	0,039
SRPX	sushi-repeat-containing protein, X-linked	cell adhesion	0,88	0,039
FLNA	filamin A, alpha (actin binding protein 280)	actin cytoskeleton organization and biogenesis /// positive regulation of I-kappaB kinase/NF-kappaB cascade	0,90	0,043
XIAP	X-linked inhibitor of apoptosis	apoptosis /// anti-apoptosis /// negative regulation of caspase activity	0,91	0,040
DYNLT3	dynein, light chain, Tctex-type 3	motor activity	0,92	0,046
TIMP1*	TIMP metallopeptidase inhibitor 1	metalloendopeptidase inhibitor activity /// positive regulation of cell proliferation	0,97	0,039
LDOC1	leucine zipper, down-regulated in cancer 1	negative regulation of cell proliferation	1,04	0,039
MID1	midline 1 (Opitz/BBB syndrome)	microtubule cytoskeleton organization and biogenesis	1,04	0,039
DDX58	DEAD (Asp-Glu-Ala-Asp) box polypeptide 58	innate immune response	1,04	0,042
CD22	CD22 molecule	immune response /// cell-cell adhesion	1,05	0,039
CRLF2	cytokine receptor-like factor 2	receptor activity	1,21	0,040
GPR87	G protein-coupled receptor 87	G-protein coupled receptor protein signaling pathway	1,33	0,042
CSF2RA*	colony stimulating factor 2 receptor, alpha, low-affinity (granulocyte-macrophage)	hematopoietin/interferon-class (D200-domain) cytokine receptor activity	1,38	0,039
MPP1	membrane protein, palmitoylated 1, 55 kDa	signal transduction /// cell projection	1,43	0,011

Genes functions are based on GO Description provided by Affymetrix, NetAffx data base (https://www.affymetrix.com/analysis/netaffx/index.affx).

*Validated by QRT-PCR.

We validated the array results by real-time PCR for selected genes (indicated with asterisks in tables 1 and 2).

### Adhesion and transmigration abilities of INV and REF cells

**Figure 2:**
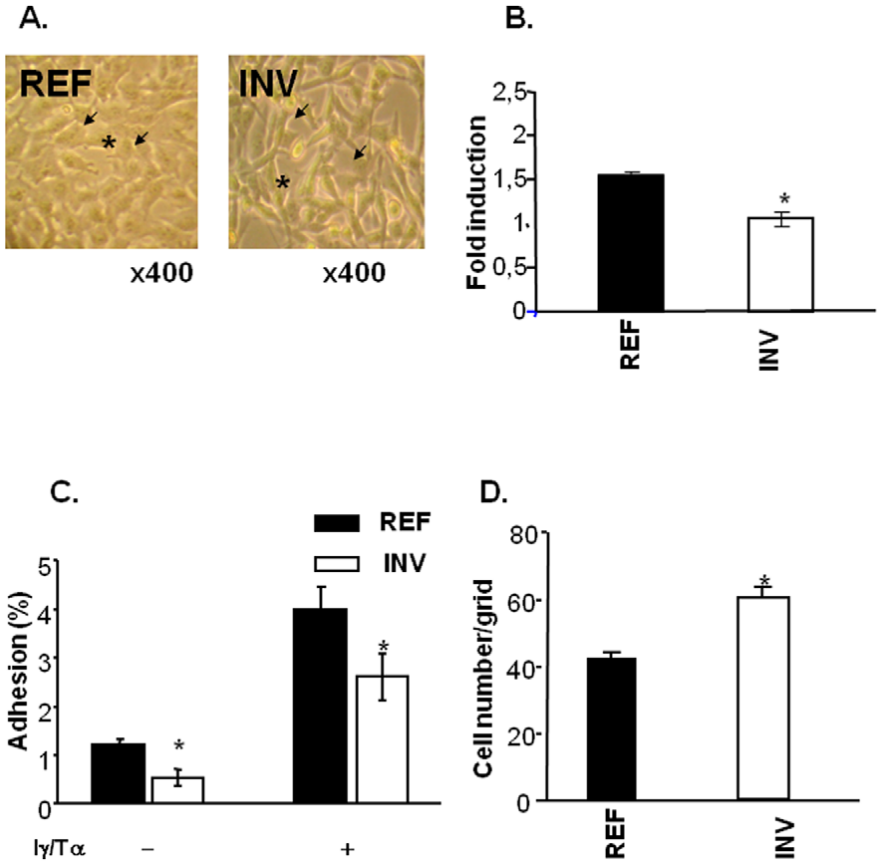
REF and INV cell adhesion and transmigration. Morphology of INV and REF cells (A) revealed by optical microscopy at ×400 magnification. Arrows indicate cell junctions and asterisks indicate cell shape. Adhesion to fibronectin (B) and hCMEC/D3 endothelial cell adhesion assay (C). Transmigration assays (D) involved growing hCMEC/D3 endothelial cells to confluence on the upper side of 8 µm insert Boyden chambers. CMFDA-stained cells that transmigrated through the endothelial cells were counted as described in “Materials and Methods”. For adhesion to fibronectin, the data were normalized to BSA adhesion. Each column shows means (± SD) of three independent experiments. *P<0.05.

The main differences between the transcriptome profiles of the two cell populations concerned molecules involved in cell-ECM and cell-cell junctions. Therefore, we tested INV and REF cells for adhesion and transmigration through endothelial cells. As observed in Fig. 2A, the morphology of INV cells in monolayer cultures differed from that of REF cells. INV but not REF monolayers cells seemed to present gaps between cells. In addition, REF cells were round whereas INV cells were fusiform.

We next tested adhesion of INV and REF cells to fibronectin and hCMEC/D3 brain microcapillary endothelial cells (Fig. 2 B and C, respectively). Consistent with findings for the transcriptomes, REF cells adhered to fibronectin whereas INV cells did not (the optical density was the same as for negative controls, equivalent to fold induction of 1) (Fig. 2B). Although both cell populations adhered to the endothelium formed by hCMEC/D3 human brain microcapillary endothelial cells, INV cells again showed a lower adhesion capacity than REF cells both in the presence or absence of cytokines (Fig. 2C) (proinflammatory cytokines have been reported to activate endothelial cells and induce tumor cell attachment to the endothelium [26]).

Next, we compared the ability of INV and REF cells to transmigrate through hCMEC/D3 endothelial cells (Fig. 2D): INV cell transmigration was 1.5 fold higher than that of REF cells (p<0.05).

### Increased metastatic sites colonization and reduced survival in INV cell-inoculated mice

**Figure 3:**
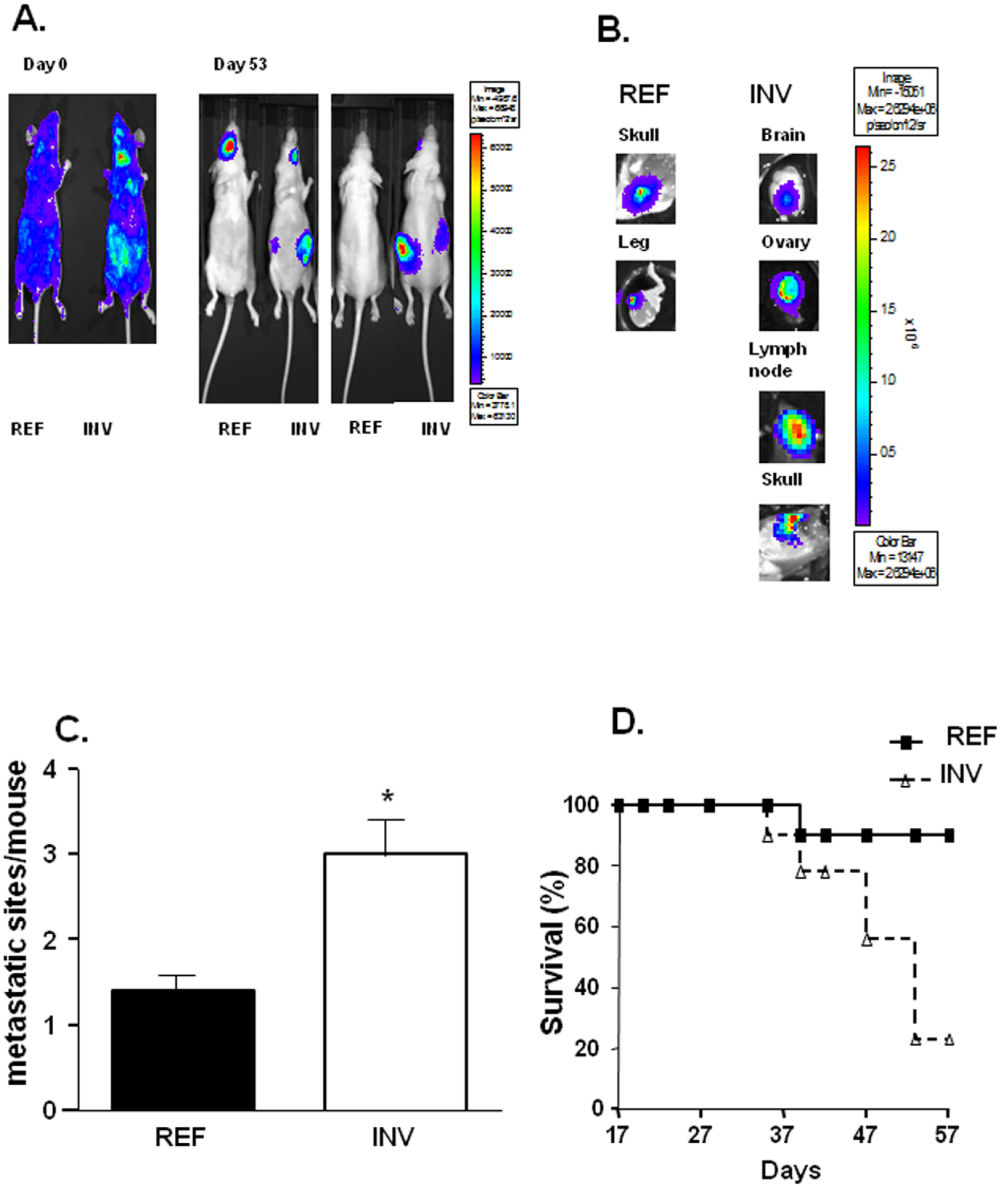
REF and INV metastasis formation. INV and REF cells (10^5^) were injected into the left ventricle of nude mice (n = 7, A). *Ex vivo* data confirm tissue metastasis from INV (right panel) and REF cells injection (left panel) (B). Quantification of metastatic sites (C). Mouse survival following injection of REF and INV cells (D). Each column shows means (±SD) of representative experiments. *P versus control<0.05.

In order to study the subclones metastasis behaviour in the whole body, Luc-transfected subclones of INV and REF (1×10^5^) were injected in the circulation (intracardiac route as described previously, [25]) of nude mice (n = 9) and their abilities to colonize secondary sites were evaluated weekly during eight weeks (Fig. 3A). Metastases were detected after two and three weeks in mice injected with INV and REF cells, respectively. Five weeks after injection, multiple metastatic sites were detected in mice inoculated with INV cells (Fig. 3A and C). In general, in each mouse inoculated with REF cells, one metastatic site was detected, mainly in bone rather if only one mouse developed lung metastasis which lead to rapid death at day 37 (data not shown. In addition, *ex vivo* imaging of various tissues after the final *in vivo* imaging mainly revealed the presence of lesions in bones for REF cells injections (legs and skull) and brain, lymph nodes and ovary for INV ones (Fig. 3B). Ovarian lesions were only found in INV cell-injected mice (4 of 9 mice) and most smooth tissue lesions were also in mice injected with INV cells. The number of metastatic sites was 2 to 3 fold higher in the INV group than in the REF group of mice (Fig. 3C). INV cell-injected mice died earlier than REF cell-injected mice; only 20% of INV as compared to 80% of the REF group survived to the end of the experiment (Fig. 3D).

### Inreased growth of INV xenografts associated with enhanced cell survival

**Figure 4:**
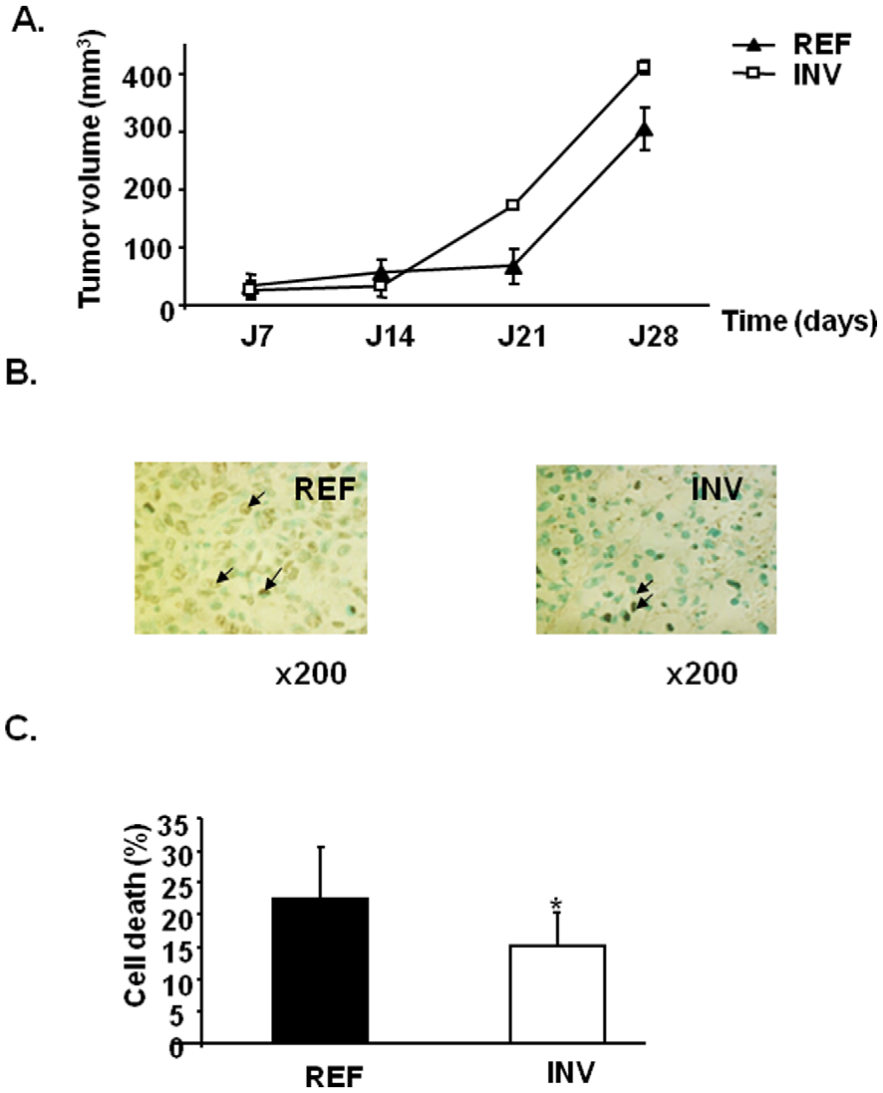
REF and INV tumor growth. INV and REF cells were injected subcutaneously near mammary gland into nude mice as described in “Materials and Methods”. After 1 week, tumors appeared in each group (A). Each point represents the mean tumor volume (mm^3^) (± SD, n = 7). Dead cells in REF and INV tumor sections were stained (B). Arrows indicate apoptotic and necrotic cells (magnification ×200). Percentage of cell death was determined as described in “Materials and Methods” (C).

**Figure 5:**
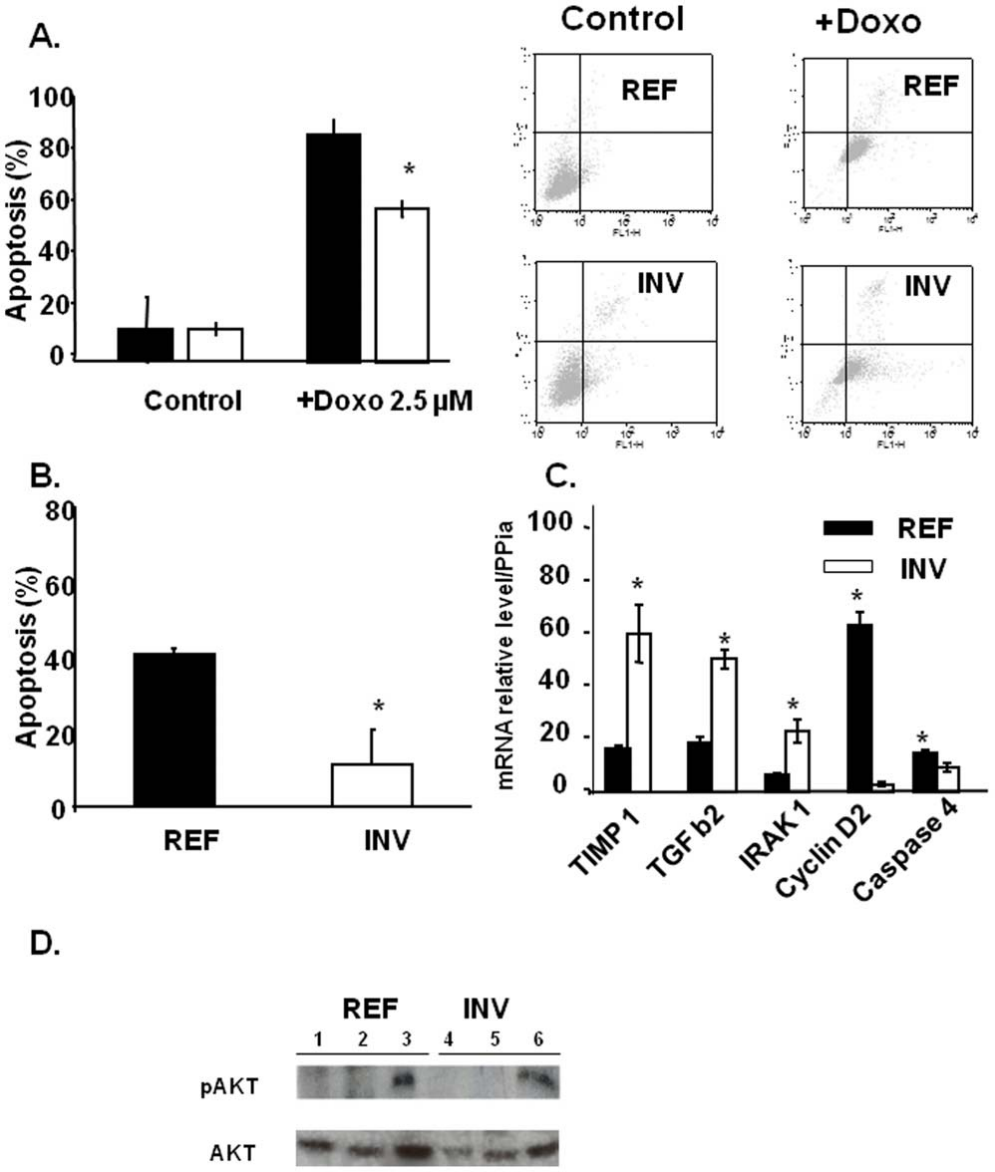
REF and INV cell death. Cells (2×10^5^) were cultured for 96 h in serum-free medium or were incubated with 2.5 µM Doxorubicin for 72 h in a serum-containing medium (A) or were cultured for 96 h in serum-free medium (B). For qRT-PCR analysis (C), total RNA (1 µg) was reverse-transcribed and the results were normalized as described above. (D) REF cells (Lanes 1 to 3) and INV cells (lanes 4 to 6) were treated (lane 1 and 4) with Serum free DMEM for 1 h or for 3 days (lanes 2 and 5) or with 10 ng/ml of IGF-1 (lanes 3 and 6), to detected AKT and pAKT by western blotting. Each column shows means (± SD) of three independent experiments. *P<0.05.

We next explored the tumorigenicity of INV and REF cells in nude mice. We injected REF or INV cells (2×10^6^) subcutaneously near the mammary gland of nude mice (Fig. 4). Both cell populations caused the development of 30 mm^3^ tumors after one week. After 21 days, the volumes of INV tumors were 2.6 fold higher than those of REF tumors (172 mm^3^ *vs* 67 mm^3^, respectively, Fig. 4A). Surprisingly, *in vitro* proliferation of INV cells was half that of REF cells after 72 h of cell culture in FCS10%/DMEM (data not shown). Thus, the faster growth of INV tumors was unlikely to have been due to a higher proliferation rate. We therefore evaluated cell survival within REF and INV tumors by *in situ* DNA fragmentation labeling with biotinylated nucleotides. This technique results in brown staining of apoptotic cell nuclei and diffuse brown staining in the cytoplasm and nuclei of necrotic cells (Fig. 4B). The numbers of dead cells were lower in INV tumors than REF tumors (15% versus 22%, *P* = 0.046; Fig. 4C). *In vitro* after 24 h of treatment with 2.5 µM doxorubicin (Fig. 5), 90% of REF and 59% of INV cells had undergone apoptosis as revealed by Annexin-V labeling (Fig. 5A). Similarly, following 96 h of serum deprivation, 40% of REF cells and only 9.5% of INV cells were found to be apoptotic (Fig. 5B). These various *in vitro* and *in vivo* analyses demonstrate that INV cells are more apoptosis-resistant than REF cells. This is consistent with the qRT-PCR and microarray evidence of up-regulation of several anti-apoptotic molecules in INV cells including *TIMP-1* ([27]), *TGF β2* ([28]), and *IRAK1* ([29]. Also, some pro-apoptotic molecules and some factors involved in drug-induced apoptosis sensitization, such as cyclin D2 and caspase 4 [30], were found to be down-regulated in INV cells. Analysis of AKT activation showed no activation when INV as well as REF cells where in serum free medium (Fig. 5D, lane 2, 3 and lane 5, 6) as compared to controls stimulated with IGF-1 (lane 1 and 4).

### Increased angiogenesis in INV xenografts

**Figure 6:**
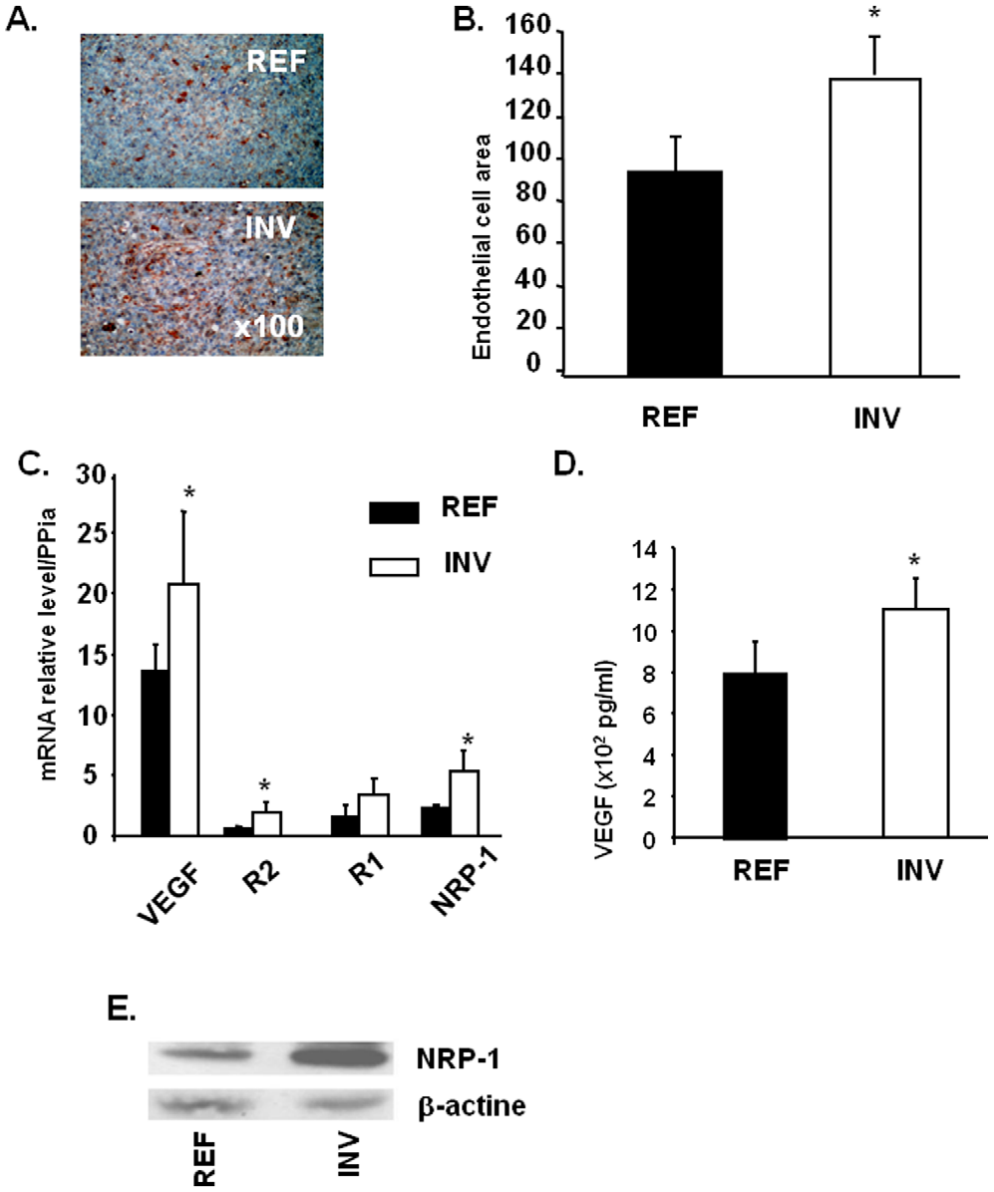
REF and INV tumor angiogenesis. Endothelial cells in REF and INV tumor sections were stained as described in “Materials and Methods”. Microvessels are revealed by brown staining (magnification ×100) and endothelial cells were quantified (B). QRT-PCR was used to quantify *VEGF*, *VEGFR2*, *VEGFR1* and *NRP-1* gene expression in INV and REF cells (C). VEGF (D) and NRP-1 (E) proteins were detected by ELISA tests and by western blotting, respectively. Each column represents a mean (± SD) of three independent experiments. *P<0.05. Gene functions from the GO Description provided by NetAffx™ Analysis Center Affymetrix (https://www.affymetrix.com/analysis/netaffx/index.affx)* Validated by QRT-PCR.

We next assessed angiogenesis in both INV and REF tumors using GSL-1 lectin to labelmouse endothelial cells (Fig. 6). Both tumor types exhibited vascular areas (Fig. 6A). The number of endothelial cells per area was significantly higher in INV than REF tumors (132 versus 94, respectively, p<0.05, Fig. 6B). Analysis of genes involved in angiogenesis including those encoding VEGF and their receptors [31]–[35] by qRT-PCR revealed stronger VEGF, VEGFR2 and NRP-1 expression in INV compared to REF cells (Fig. 6C). The over-production of VEGF and NRP-1 proteins was confirmed by ELISA tests for VEGF (Fig. 6D) and western blot analysis for NRP-1 (Fig. 6E).

## Discussion

We report that breast cancer cell invasion through Matrigel resulted in the generation of an aggressive subpopulation (INV) with a stable malignant genotypic/phenotypic profile. The main characteristic of these cells was loss of adhesion to ECM, and in particular fibronectin and endothelial cells. Accordingly, in nude mice, INV cells were more competent than the control cells (REF) for extravasation through the endothelium to colonise tissues and form metastases. Metastases formed by INV cells were mainly found in various soft tissue (brain, ovary, lymph node and bone) as compared to REF ones (Exclusively in bones). This result was in accordance with previous work of Jenkins et al [36] which demonstrated that dissemination in other sites revealed a hallmark of increased metastatic potential. This indicates that the ECM is sufficient to influence the enrichment of a stable metastatic subpopulation. We also found that INV cells failed to induce significant platelet adhesion and/or aggregation (data not shown), a process previously reported to be involved in metastasis processes [37]. Previously, cell migration was reported to result from a balance between cell adhesion and de-adhesion, both acquired for motility. Indeed, strong adhesion would tightly attach cancer cells to the substrate and prevent their movement. Reduced cell adhesion to both ECM and endothelial cells, observed for our INV subpopulation, is consistent with their enhanced migration ability, and their capacity to cross the endothelial barrier to invade secondary organs and form metastases. Thus, we found that the cells obtained by transmigration through an extracellular matrix displayed a loss of adhesion behavior. Studies on breast cancer progression indicate that loss of cadherin or cell polarity and the acquisition of a mesenchymal phenotype are associated with an increase in invasiveness [38]. Accordingly, our INV cells, the invasive subpopulation, had a fusiform shape with large spaces between cells and thus resembled mesenchymal cells. Also, we found weak expression of *OCLN* and strong expression of *TGFβ2*, an association which was previously demonstrated to be linked to the breast cancer epithelial mesenchymal transition (EMT) [39]. Thus, our results support the notion that the basement membrane may also contribute to the EMT leading to plasticity of metastatic cells.

Using GSEA MsigDB [40] data bases, we found that the functions of 38% of down-regulated and 47% of up-regulated genes in INV are known (see also Fig. S1). Most are involved in the immune/inflammatory response, cell cycle (M phase), adhesion and dorsal root ganglion. Accordingly, 44% of the known down-regulated genes in INV cells are involved in adhesion and cell-cell junction (*FERMT3*, *GABRA3*, *OCLN*, *AMIGO2*, *SCARF1*, *ASAM*, *PCDHB2*).

Many (25%) of the genes more strongly expressed in INV than REF cells, are linked to cell survival by negative regulation of apoptosis. INV cells also over-expressed the proangiogenic factor (VEGF) and its receptor NRP-1, both involved in the autocrine survival of cells and microvessel permeability, processes required for cell extravasation [41]. Other genes including *IRAK1*, *NKAP*, *FLNA*, *XIAP* and *TGFß2* were also up-regulated: *XIAP* and *TGFβ2* are associated with the NFKB pathways possibly being pivotal potential signaling targets [42], [43]. To note, also the implication of UPA, UPAR and PAR-I in the NFKB pathway and that acted in concert with MMP-9 to enhance INV cell invasion. Thus, further studies on the NFKB signaling in these cell would seem interesting to undergo. Some pro-apoptotic genes were down-regulated in INV cells. However, important pro-apoptotic gene as for example, BCL2 was not found to be upregulated, (Fig. S2). These include rather *CASP4* and cyclin D2, previously reported to be involved in genistein and doxorubicin sensitization, respectively [44], [30]. The down-regulation of cyclin D2 in INV cells suggests neosis, an important process that mediates tumor cell resistance to apoptosis, heterogeneity and continuity [45]. These findings are in agreement with reports of the critical role of survival during the metastatic process of tumor cells [46]. Further, AKT analysis showed no differences between the INV and REF cells, suggesting that several survival genes mentioned above acted in concert to increase resistance to death. Interestingly, *in vivo*, we only observed small differences in cell death between INV cell-derived tumors and REF tumors, suggesting that the observed difference in tumor volumes might result from a dormancy exit. Indeed, several reports reveal the participation of angiogenesis in the exit from dormancy by inhibiting apoptosis [47]. The *in vitro* identification of anti-apoptotic or survival targets in this work constitutes an interesting and original finding that could lead to better treat aggressive sub populations. In particular, it could be a good strategy to prevent cancer relapse and to better investigate tumor dormancy in term of therapy as well as diagnosis. In basis of these first results, further investigation should address preclinal models to indentify targets in order to treat and also survey long-term cancer survivors minimizing the risk of cancer recurrence.

### Conclusions

We demonstrated that using only basement membrane extracellular matrix it is possible to obtain a stable metastatic population without using expensive and longer *in vivo* procedures. Our results suggest that future strategies for anti-metastatic therapies should target molecules involved in angiogenesis and survival rather than in cell adhesion. We report here the genetic, phenotypic and functional characterization of invasive (INV) cancer cell population. We believe that such cells will provide unique cellular models for *in vitro* screening of anti-metastatic drugs with clinical potential.

## Supporting Information

Figure S1 **Gene expression analysis of REF and INV cells.** Down-regulated genes (A) and up-regulated genes (B) were determined using Affymetrix Human Gene 1.0ST arrays as described in “Materials and Methods”.
(TIF)

Figure S2 ***BCL2*** **mRNA quantification in REF and INV cells.** Total RNA (1 µg) was reverse-transcribed using MMLV RT and subjected to qRT-PCR as described in Material and Methods.
(TIF)

Table S1 **Complete list of genes that are down- and up-regulated in INV cells relative to REF cells.** Gene functions are taken from the GO Description provided by NetAffx™ Analysis Center Affymetrix (https://www.affymetrix.com/analysis/netaffx/index.affx). * Validated by qRT-PCR.
(DOC)
