# Supplementary figures and images for: Invading Basement Membrane Matrix Is Sufficient for MDA-MB-231 Breast Cancer Cells to Develop a Stable In Vivo Metastatic Phenotype
Source: PLoS One. 2011 Aug 15;6(8):e23334. doi: 10.1371/journal.pone.0023334 (PMC3156115; doi:10.1371/journal.pone.0023334)

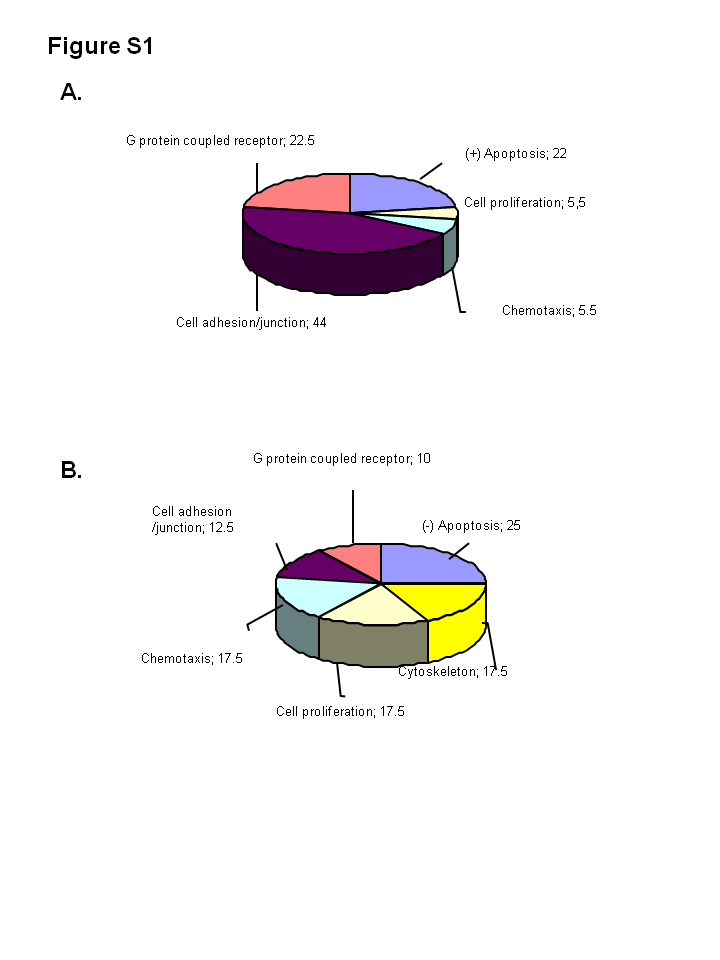

Supplement: Figure S1 — Gene expression analysis of REF and INV cells. Down-regulated genes (A) and up-regulated genes (B) were determined using Affymetrix Human Gene 1.0ST arrays as described in “Materials and Methods”. (TIF) [file pone.0023334.s001.tif]

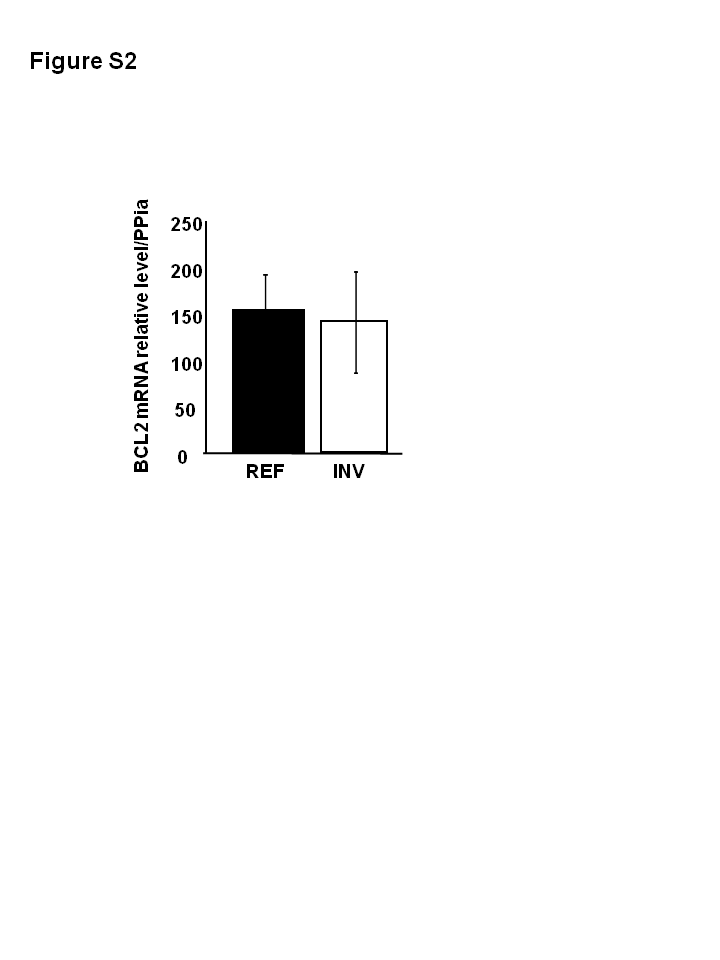

Supplement: Figure S2 — BCL2 mRNA quantification in REF and INV cells. Total RNA (1 µg) was reverse-transcribed using MMLV RT and subjected to qRT-PCR as described in Material and Methods. (TIF) [file pone.0023334.s002.tif]
