# Supplementary material for: Invading Basement Membrane Matrix Is Sufficient for MDA-MB-231 Breast Cancer Cells to Develop a Stable In Vivo Metastatic Phenotype
Source: PLoS One. 2011 Aug 15;6(8):e23334. doi: 10.1371/journal.pone.0023334 (PMC3156115; doi:10.1371/journal.pone.0023334)
Supplement: Table S1 — Complete list of genes that are down- and up-regulated in INV cells relative to REF cells. Gene functions are taken from the GO Description provided by NetAffx™ Analysis Center Affymetrix (https://www.affymetrix.com/analysis/netaffx/index.affx). * Validated by qRT-PCR. (DOC) [file pone.0023334.s003.doc]

| **Probeset ID** | **Gene Symbol** | **Gene Title** | **log fold** | **Adjusted p-value** |
| --- | --- | --- | --- | --- |
| **Down - regulated (Inv vs Ref)** | | |  |  |
| 8026490 | UCA1 | urothelial cancer associated 1 | -2,48 | 0,006 |
| 7953200 | CCND2 * | cyclin D2 | -2,25 | 0,039 |
| 7940869 | FERMT3 * | fermitin family homolog 3 (Drosophila) | -1,70 | 0,039 |
| 8123080 | SYTL3 | synaptotagmin-like 3 | -1,61 | 0,021 |
| 8162502 | FBP1 | fructose-1,6-bisphosphatase 1 | -1,55 | 0,039 |
| 8175696 | GABRA3 | gamma-aminobutyric acid (GABA) A receptor, alpha 3 | -1,38 | 0,022 |
| 8008185 | ABI3 | ABI gene family, member 3 | -1,28 | 0,039 |
| 8111941 | HMGCS1 | 3-hydroxy-3-methylglutaryl-Coenzyme A synthase 1 (soluble) | -1,16 | 0,046 |
| 8105908 | OCLN | occludin | -1,12 | 0,040 |
| 8107594 | SNCAIP | synuclein, alpha interacting protein | -1,12 | 0,039 |
| 8170553 | MAGEA6 /// MAGEA3 | melanoma antigen family A, 6 / A, 3 | -1,10 | 0,040 |
| 7987439 | GPR176 | G protein-coupled receptor 176 | -1,07 | 0,040 |
| 7962579 | AMIGO2 | adhesion molecule with Ig-like domain 2 | -1,03 | 0,046 |
| 7988467 | FBN1 | fibrillin 1 | -1,00 | 0,039 |
| 8016532 | GNGT2 | guanine nucleotide binding protein (G protein), gamma transducing activity polypeptide 2 | -0,99 | 0,039 |
| 7952341 | ASAM | adipocyte-specific adhesion molecule | -0,97 | 0,042 |
| 8105506 | ZSWIM6 | zinc finger, SWIM-type containing 6 | -0,97 | 0,044 |
| 8106280 | HMGCR | 3-hydroxy-3-methylglutaryl-Coenzyme A reductase | -0,93 | 0,050 |
| 7931930 | PRKCQ | protein kinase C, theta | -0,92 | 0,040 |
| 8011114 | SCARF1 | scavenger receptor class F, member 1 | -0,91 | 0,039 |
| 7940565 | FADS2 | fatty acid desaturase 2 | -0,91 | 0,040 |
| 8053648 | KRCC1 | lysine-rich coiled-coil 1 | -0,88 | 0,040 |
| 7983650 | SLC27A2 | solute carrier family 27 (fatty acid transporter), member 2 | -0,86 | 0,039 |
| 7989094 | NEDD4 | neural precursor cell expressed, developmentally down-regulated 4 | -0,86 | 0,040 |
| 8048171 | DKFZp434H1419 | hypothetical protein DKFZp434H1419 | -0,82 | 0,040 |
| 8007212 | STAT5A | signal transducer and activator of transcription 5A | -0,81 | 0,047 |
| 8059413 | DOCK10 | dedicator of cytokinesis 10 | -0,81 | 0,040 |
| 7951372 | CASP4 * | caspase 4, apoptosis-related cysteine peptidase | -0,79 | 0,046 |
| 8108683 | PCDHB2 | protocadherin beta 2 | -0,74 | 0,040 |
| 8105077 | CARD6 | caspase recruitment domain family, member 6 | -0,73 | 0,043 |
| 8005132 | MEIS3 | Meis homeobox 3 | -0,72 | 0,042 |
| 7939676 | KIAA0652 | KIAA0652 | -0,72 | 0,045 |
| 7988093 | TP53BP1 | tumor protein p53 binding protein 1 | -0,72 | 0,046 |
| 7982957 | MGA | MAX gene associated | -0,66 | 0,042 |
| 7944722 | UBASH3B | ubiquitin associated and SH3 domain containing, B | -0,64 | 0,043 |
| 8007228 | ATP6V0A1 | ATPase, H+ transporting, lysosomal V0 subunit a1 | -0,61 | 0,046 |
| 8094228 | BST1 | bone marrow stromal cell antigen 1 | -0,61 | 0,047 |
| 8105191 | PARP8 | poly (ADP-ribose) polymerase family, member 8 | -0,58 | 0,048 |
| **Up- regulated (Inv vs Ref)** | | |  |  |
| 8166072 | TMSB4X /// TMSL3 /// TMSL1 /// TMSL2 /// TMSL6 | thymosin beta 4, X-linked /// thymosin-like 3 /// thymosin-like 1 (pseudogene) /// thymosin-like 2 (pseudogene) /// thymosin-like 6 (pseudogene) | 0,56 | 0,050 |
| 8027556 | LRP3 | low density lipoprotein receptor-related protein 3 | 0,57 | 0,047 |
| 8169249 | MID2 | midline 2 | 0,58 | 0,049 |
| 8167449 | PLP2 | proteolipid protein 2 (colonic epithelium-enriched) | 0,61 | 0,048 |
| 8166826 | USP9X | ubiquitin specific peptidase 9, X-linked | 0,61 | 0,047 |
| 7909789 | TGFB2 * | transforming growth factor, beta 2 | 0,62 | 0,048 |
| 8172478 | OTUD5 | OTU domain containing 5 | 0,63 | 0,046 |
| 8175755 | CETN2 | centrin, EF-hand protein, 2 | 0,64 | 0,046 |
| 8074991 | GGT5 | gamma-glutamyltransferase 5 | 0,64 | 0,048 |
| 8067007 | TMSB4X /// TMSL2 /// TMSL6 /// TMSL3 | thymosin beta 4, X-linked /// thymosin-like 2 (pseudogene) /// thymosin-like 6 (pseudogene) /// thymosin-like 3 | 0,64 | 0,046 |
| 8170479 | LOC203547 | hypothetical protein LOC203547 | 0,65 | 0,044 |
| 8045075 | GPR17 | G protein-coupled receptor 17 | 0,65 | 0,042 |
| 8171229 | PNPLA4 | patatin-like phospholipase domain containing 4 | 0,66 | 0,040 |
| 8175871 | L1CAM * | L1 cell adhesion molecule | 0,66 | 0,046 |
| 8034416 | RPL10 | ribosomal protein L10 | 0,66 | 0,043 |
| 8171802 | ACOT9 | acyl-CoA thioesterase 9 | 0,67 | 0,044 |
| 8175023 | ZDHHC9 | zinc finger, DHHC-type containing 9 | 0,68 | 0,042 |
| 8168868 | ARMCX1 | armadillo repeat containing, X-linked 1 | 0,69 | 0,043 |
| 8170891 | GDI1 | GDP dissociation inhibitor 1 | 0,69 | 0,045 |
| 8167287 | PORCN | porcupine homolog (Drosophila) | 0,70 | 0,042 |
| 8167656 | MAGED1 | melanoma antigen family D, 1 | 0,70 | 0,048 |
| 8171516 | RBBP7 * | retinoblastoma binding protein 7 | 0,70 | 0,042 |
| 8170166 | HTATSF1 | HIV-1 Tat specific factor 1 | 0,70 | 0,040 |
| 8175710 | CSAG2 /// CSAG3 /// CSAG1 | CSAG family, member 2 /// CSAG family, member 3 /// chondrosarcoma associated gene 1 | 0,71 | 0,049 |
| 8175647 | CD99L2 | CD99 molecule-like 2 | 0,71 | 0,040 |
| 8167013 | PHF16 | PHD finger protein 16 | 0,72 | 0,042 |
| 8157905 | FAM125B | family with sequence similarity 125, member B | 0,72 | 0,050 |
| 8167790 | TSR2 | TSR2, 20S rRNA accumulation, homolog (S. cerevisiae) | 0,72 | 0,046 |
| 8172244 | FUNDC1 | FUN14 domain containing 1 | 0,73 | 0,043 |
| 8166805 | ATP6AP2 | ATPase, H+ transporting, lysosomal accessory protein 2 | 0,73 | 0,040 |
| 8171381 | FANCB | Fanconi anemia, complementation group B | 0,73 | 0,040 |
| 8170326 | FMR1 | fragile X mental retardation 1 | 0,73 | 0,048 |
| 8169969 | PHF6 | PHD finger protein 6 | 0,73 | 0,050 |
| 8175977 | IRAK1 * | interleukin-1 receptor-associated kinase 1 | 0,73 | 0,042 |
| 8167347 | SUV39H1 | suppressor of variegation 3-9 homolog 1 (Drosophila) | 0,73 | 0,040 |
| 8172827 | JARID1C | jumonji, AT rich interactive domain 1C | 0,74 | 0,040 |
| 8175835 | BCAP31 | B-cell receptor-associated protein 31 | 0,74 | 0,040 |
| 8172504 | GRIPAP1 | GRIP1 associated protein 1 | 0,74 | 0,040 |
| 8173086 | FGD1 | FYVE, RhoGEF and PH domain containing 1 | 0,74 | 0,040 |
| 8154727 | LOC138412 | solute carrier family 25 (mitochondrial carrier; adenine nucleotide translocator), member 6, pseudogene | 0,74 | 0,040 |
| 8174737 | NKAP | NFKB activating protein | 0,75 | 0,040 |
| 8172520 | TFE3 | transcription factor binding to IGHM enhancer 3 | 0,76 | 0,048 |
| 8168749 | SRPX2 | sushi-repeat-containing protein, X-linked 2 | 0,76 | 0,040 |
| 8174340 | RBM41 | RNA binding motif protein 41 | 0,76 | 0,042 |
| 8170390 | LOC727913 /// IDS | similar to iduronate 2-sulfatase (Hunter syndrome) /// iduronate 2-sulfatase | 0,76 | 0,042 |
| 8173232 | FAM123B | family with sequence similarity 123B | 0,77 | 0,039 |
| 8172119 | MED14 | mediator complex subunit 14 | 0,77 | 0,042 |
| 8166382 | MBTPS2 | membrane-bound transcription factor peptidase, site 2 | 0,77 | 0,045 |
| 7997139 | CALB2 | calbindin 2 | 0,77 | 0,040 |
| 8166455 | PRDX4 | peroxiredoxin 4 | 0,77 | 0,043 |
| 8166098 | RAB9A | RAB9A, member RAS oncogene family | 0,77 | 0,040 |
| 8171392 | ASB9 | ankyrin repeat and SOCS box-containing 9 | 0,77 | 0,040 |
| 8165794 | CD99 | CD99 molecule | 0,77 | 0,042 |
| 8167165 | ARAF | v-raf murine sarcoma 3611 viral oncogene homolog | 0,78 | 0,040 |
| 8175593 | IDS | iduronate 2-sulfatase | 0,78 | 0,040 |
| 8175052 | AIFM1 | apoptosis-inducing factor, mitochondrion-associated, 1 | 0,79 | 0,040 |
| 8167006 | RP2 | retinitis pigmentosa 2 (X-linked recessive) | 0,79 | 0,040 |
| 8089911 | HCLS1 | hematopoietic cell-specific Lyn substrate 1 | 0,79 | 0,049 |
| 8167369 | HDAC6 | histone deacetylase 6 | 0,79 | 0,039 |
| 8167854 | APEX2 | APEX nuclease (apurinic/apyrimidinic endonuclease) 2 | 0,79 | 0,040 |
| 8169920 | RBMX2 | RNA binding motif protein, X-linked 2 | 0,80 | 0,042 |
| 8172358 | UXT | ubiquitously-expressed transcript | 0,80 | 0,045 |
| 8167322 | WDR13 | WD repeat domain 13 | 0,81 | 0,040 |
| 8171041 | VAMP7 | vesicle-associated membrane protein 7 | 0,81 | 0,046 |
| 8167069 | UBA1 | ubiquitin-like modifier activating enzyme 1 | 0,81 | 0,040 |
| 8170850 | EMD | emerin | 0,82 | 0,039 |
| 8171013 | VBP1 | von Hippel-Lindau binding protein 1 | 0,83 | 0,040 |
| 8171111 | SLC25A6 /// LOC138412 | solute carrier family 25 (mitochondrial carrier; adenine nucleotide translocator), member 6 /// solute carrier family 25 (mitochondrial carrier; adenine nucleotide translocator), member 6, pseudogene | 0,83 | 0,039 |
| 8166500 | ZFX | zinc finger protein, X-linked | 0,84 | 0,040 |
| 8166140 | MOSPD2 | motile sperm domain containing 2 | 0,85 | 0,040 |
| 8175302 | FAM127B | family with sequence similarity 127, member B | 0,86 | 0,042 |
| 8170906 | FAM50A | family with sequence similarity 50, member A | 0,86 | 0,040 |
| 8172043 | SRPX | sushi-repeat-containing protein, X-linked | 0,88 | 0,039 |
| 8169811 | OCRL | oculocerebrorenal syndrome of Lowe | 0,88 | 0,042 |
| 8175811 | FAM58A | family with sequence similarity 58, member A | 0,88 | 0,039 |
| 8177011 | ASMTL | acetylserotonin O-methyltransferase-like | 0,89 | 0,044 |
| 8166335 | PDHA1 | pyruvate dehydrogenase (lipoamide) alpha 1 | 0,90 | 0,039 |
| 8176026 | FLNA | filamin A, alpha (actin binding protein 280) | 0,90 | 0,043 |
| 8167305 | EBP | emopamil binding protein (sterol isomerase) | 0,91 | 0,039 |
| 8169742 | XIAP | X-linked inhibitor of apoptosis | 0,91 | 0,040 |
| 8107563 | PRR16 | proline rich 16 | 0,92 | 0,046 |
| 8172035 | DYNLT3 | dynein, light chain, Tctex-type 3 | 0,92 | 0,046 |
| 8174820 | C1GALT1C1 | C1GALT1-specific chaperone 1 | 0,93 | 0,039 |
| 8063923 | SLCO4A1 | solute carrier organic anion transporter family, member 4A1 | 0,96 | 0,040 |
| 8171493 | CTPS2 | CTP synthase II | 0,96 | 0,045 |
| 8174654 | KLHL13 | kelch-like 13 (Drosophila) | 0,97 | 0,040 |
| 8167185 | TIMP1 * | TIMP metallopeptidase inhibitor 1 | 0,97 | 0,039 |
| 8175539 | LDOC1 | leucine zipper, down-regulated in cancer 1 | 1,04 | 0,039 |
| 8171297 | MID1 | midline 1 (Opitz/BBB syndrome) | 1,04 | 0,039 |
| 8122222 | PDE7B | phosphodiesterase 7B | 1,04 | 0,048 |
| 8160559 | DDX58 | DEAD (Asp-Glu-Ala-Asp) box polypeptide 58 | 1,04 | 0,042 |
| 8027837 | CD22 | CD22 molecule | 1,05 | 0,039 |
| 8171105 | CRLF2 | cytokine receptor-like factor 2 | 1,21 | 0,040 |
| 8091515 | GPR87 | G protein-coupled receptor 87 | 1,33 | 0,042 |
| 8165735 | CSF2RA * | colony stimulating factor 2 receptor, alpha, low-affinity (granulocyte-macrophage) | 1,38 | 0,039 |
| 8176174 | MPP1 | membrane protein, palmitoylated 1, 55kDa | 1,43 | 0,011 |

* confirmed by qRT-PCR

List of 134 differentially expressed genes after applying filter for adjusted p-value (≤0.05) and making the list non-redundant.
